# Influence of HER2-low and HER2-zero status on pathologic complete response and survival in triple-negative breast cancer: a meta-analysis

**DOI:** 10.3389/fonc.2025.1631125

**Published:** 2025-09-11

**Authors:** Yu Qin, Chenchen Pu, Yuping Fan, Kepeng Zhu

**Affiliations:** The Affiliated Nanchong Central Hospital of North Sichuan Medical College, Nanchong, Sichuan, China

**Keywords:** triple-negative breast cancer, HER2 status, pathological complete response, overall survival, meta-analysis

## Abstract

**Objective:**

To delve into the influence of different status of human epidermal growth factor receptor 2 (HER2) on the long-term survival of patients suffering from triple-negative breast cancer (TNBC), as well as the pathological complete response (pCR) following neoadjuvant therapy (NAT) via meta-analysis.

**Methods:**

A computer search in the Embase, PubMed, Web of Science, and Cochrane Library databases was executed up to January 13, 2025, to collect studies related to HER2 status in TNBC patients. The articles were screened per the inclusion and exclusion criteria. The required data were extracted. The study quality was appraised by means of the Newcastle-Ottawa Scale, and statistical analysis was carried out utilizing Stata 15.0 software.

**Results:**

36 studies involving 54,277 patients with TNBC were included. According to the meta-analysis, the pCR rate after NAT was more notable in the HER2-zero group compared to the HER2-low group (*RR* = 0.90, 95%*CI*: 0.86-0.93, *P <* 0.001). Regarding overall survival (OS), HER2-low patients exhibited a better prognosis (*HR* = 0.93, 95%*CI*: 0.90-0.97, *P* < 0.001). For disease-free survival, breast cancer-specific survival, and recurrence-free survival, HER2-low patients might experience an enhanced prognosis. However, the results did not exhibit statistically significant. The sensitivity analysis confirmed the robustness of the meta-analysis results. No publication bias existed in studies on each outcome indicator.

**Conclusion:**

HER2 status is essential for the prognostic assessment of TNBC patients, particularly in predicting pCR and OS outcomes.

**Systematic review registration:**

https://www.crd.york.ac.uk/prospero/, identifier PROSPERO CRD-420250642369.

## 1 Introduction

Globally, breast cancer (BC) ranks among the most prevalent malignant tumors in women. Its disease burden has remained a leading concern in oncology. As per GLOBOCAN 2022, the global incidence of new BC cases is around 2.3 million, ranking first in cancer incidence worldwide (1). Triple-negative BC (TNBC) is an aggressive subtype of BC, characterized by unique molecular features. It is named for the absence of estrogen receptors (ER), progesterone receptors (PR), and human epidermal growth factor receptor 2 (HER2) gene amplification. TNBC constitutes roughly 15%-20% of all BC cases and exhibits distinct clinical and pathological characteristics that set it apart from other subtypes. On the one hand, it demonstrates an inherent resistance to endocrine therapy and anti-HER2 targeted treatments, resulting in chemotherapy as the primary treatment option in clinical practice. On the other hand, this subtype is linked to a tendency for early recurrence and metastasis, leading to a poorer prognosis (2). In addition, TNBC is highly heterogeneous, with notable differences in the response of different subtypes to treatment.

HER2 is a transmembrane protein on the cell surface, crucial in regulating cell growth and division. In BC, tumors that overexpress HER2 exhibit greater invasiveness compared to hormone receptor-positive types. However, the application of targeted therapies against HER2, like trastuzumab, often results in favorable treatment outcomes and prognosis for HER2-positive BC (3). According to the latest testing guidelines for HER2 in BC, HER2-zero is defined as no HER2 protein expression detected by immunohistochemistry (IHC) and no gene amplification confirmed by *in situ* hybridization (ISH). HER2-low is defined by low HER2 protein expression (IHC 1+ or IHC 2+ with negative ISH). Nevertheless, it does not meet the standard for traditional HER2-positive (IHC 3+ or positive ISH) (4). Despite the traditional understanding that TNBC is marked by negative expression of HER2, recent studies have identified instances of low or borderline positive HER2 expression in some patients diagnosed with TNBC (5). This phenomenon has prompted investigators to reconsider the clinical importance of HER2 status in patients suffering from TNBC. Given the noticeable efficacy of HER2-targeted therapies in non-TNBC subtypes, it is vital to examine the clinical implications of HER2 status in patients suffering from TNBC. Existing research has demonstrated a marked connection of HER2 status with both the pathological complete response (pCR) and overall survival (OS) in patients suffering from BC (6). However, the effects of HER2 status on prognosis remain contentious, especially within the population of TNBC patients undergoing neoadjuvant therapy (NAT) (7, 8). Thus, this study intends to execute a systematic review and meta-analysis to thoroughly appraise the influence of HER2 status on the prognosis of TNBC patients, aiming to offer evidence-based support for TNBC treatment.

## 2 Materials and methods

This study was written per the Preferred Reporting Items for Systematic Reviews and Meta-Analyses (PRISMA) statement (9) and had been registered with the International Prospective Register of Systematic Reviews (PROSPERO, ID: CRD-420250642369).

### 2.1 Literature search

A computer search in the Embase, PubMed, Web of Science, and Cochrane Library databases was executed to collect articles related to HER2 status in TNBC patients, covering the period from database inception to January 13, 2025. The search terms included “Triple Negative Breast Cancer”, “ER Negative PR Negative HER2 Negative Breast Cancer”, “ErbB-2 Receptor”, “epidermal growth factor receptor 2”, and etc. Relevant studies were retrieved through a systematic search that combined subject headings and free terms. The specific search strategy is listed in the attached table.

### 2.2 Inclusion and exclusion criteria

Inclusion criteria: (i) The study participants were patients with TNBC confirmed by clinical pathological biopsy, regardless of the method of tissue biopsy or tumor staging; (ii) The study design was a cohort study; (iii) The exposure factor was defined as HER2-low status; (iv) Studies mentioned one of the following outcome measures were incorporated: pCR, OS, disease-free survival (DFS), progression-free survival (PFS), breast cancer-specific survival (BCSS), and recurrence-free survival (RFS).

Exclusion criteria: (i) study types like reviews, meta-analyses, case reports, and conference abstracts; (ii) inability to obtain or convert relevant data; (iii) non-English publications; (iv) full text not accessible.

### 2.3 Study screening and data extraction

The literature screening and data extraction were executed independently by two investigators, with the results cross-verified. In cases of discrepancies, resolution was achieved via discussion or by consulting a third investigator. Using Endnote X9 for literature screening, we first conducted a preliminary review of titles and abstracts to remove clearly ineligible papers. A full-text review was then executed to determine final inclusion in the study. Microsoft Excel spreadsheet tools were leveraged to create a data extraction table that extracted: (i) basic information about included studies, including authors, publication time, study sample size, and follow-up time; (ii) relevant factors to appraise the quality of the studies; and (iii) outcome measures and univariate and multivariate analysis results.

### 2.4 Risk of bias assessment

Two investigators independently appraised the included articles by means of the Newcastle-Ottawa Scale (NOS) based on cohort research and resolved any discrepancies through discussion. This scale is a tool specifically designed to appraise the quality of non-randomized studies (like cohort and case-control studies), known for its high reliability and broad applicability. It encompassed three modules that assess relevant factors for study population selection, inter-group comparability, and outcome evaluation. There were 8 questions, each rated from 0 to 2 points, with a maximum score of 9 points. Higher scores suggested better research quality. According to the scoring results, studies were categorized into three levels: high quality (7–9 points), moderate quality (4–6 points), and low quality (0–3 points) (10).

### 2.5 Statistical analysis

Data were statistically analyzed by means of Stata 15.0 software. The HR values and 95% confidence intervals (CIs) for the outcome measures were processed utilizing logarithmic transformation. For studies providing only survival curves, graphic extraction tools were utilized to obtain estimated HR values and 95%CIs (11). *P*<0.05 was considered to be statistically significant. Cochran’s Q test and *I^2^* statistic were employed to quantitatively examine the level of heterogeneity across the included articles. If *I^2^* ≥ 50% and *P* ≤ 0.1, it was deemed that there existed a noticeable heterogeneity among the studies. Consequently, a random-effects model (REM) was employed. Conversely, a fixed-effects model (FEM) was utilized. Subgroup analyses were implemented based on different continents and tumor staging, to further elucidate the sources of heterogeneity. We executed a sensitivity analysis utilizing the leave-one-out method on the studies included, aiming to assess the stability of the results. By creating a funnel plot and, in conjunction with Egger’s test, publication bias was examined. *P* > 0.05 demonstrated no remarkable publication bias. If publication bias was detected, an imputation method was employed for calibration.

## 3 Results

### 3.1 Literature screening results

**Figure 1:**
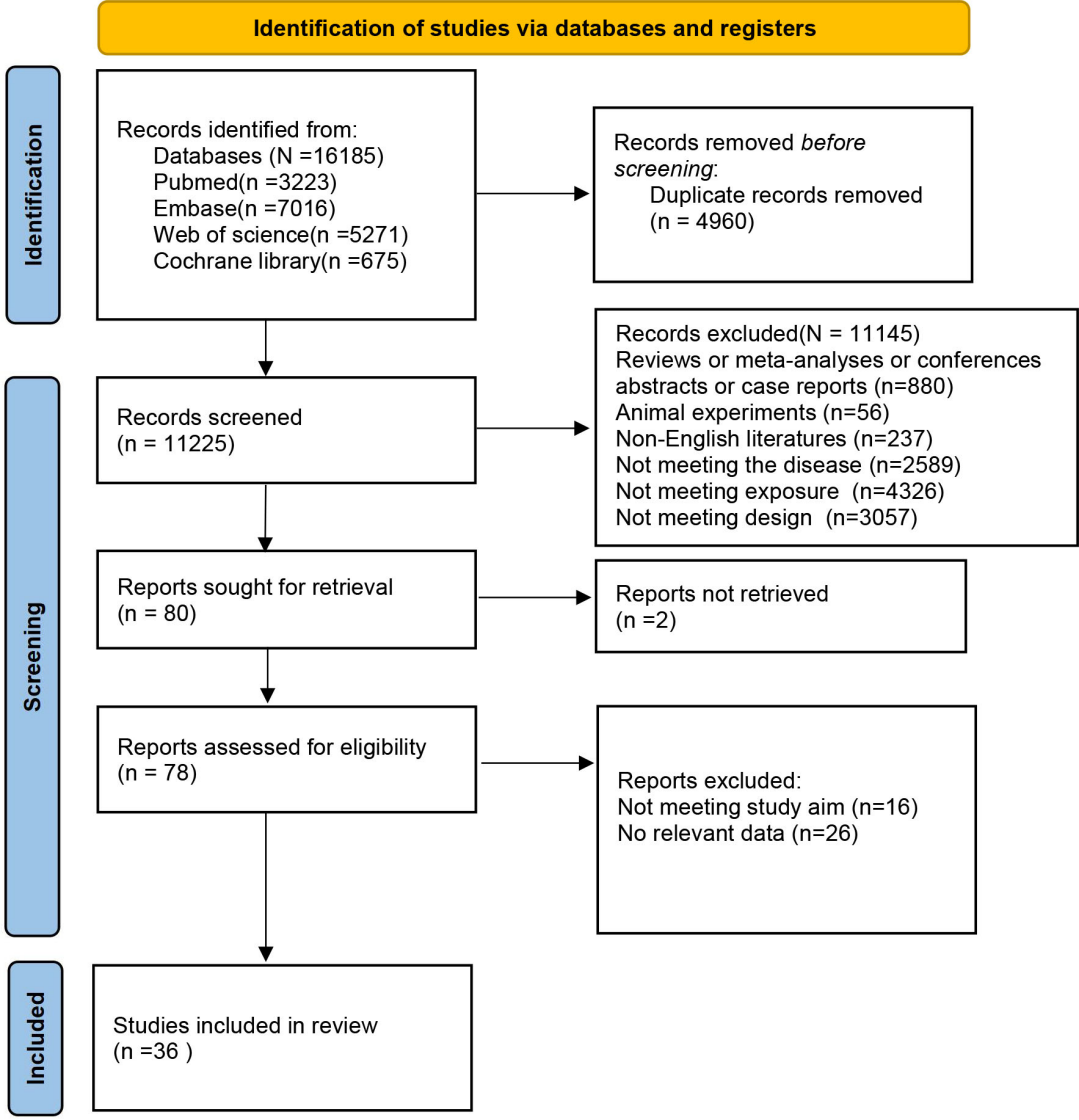
PRISMA flowchart of study selection.

Using the predefined retrieval strategy, 16,185 articles were identified from the databases. After removing 4,960 duplicate records, 11,225 articles proceeded to the initial screening phase. Following a review of titles and abstracts, 11,145 ineligible papers were deleted. Finally, 80 articles advanced to the secondary screening stage. A detailed reading of these full texts resulted in the removal of 16 articles due to misalignment with research objectives and an additional 26 articles for no relevant data. Consequently, 36 retrospective cohort studies (12–47) were included in the meta-analysis. The literature selection process and results are displayed in **Figure 1**.

### 3.2 Basic characteristics of included studies and risk of bias assessment

36 studies included 54,277 TNBC patients, with 21,403 HER2-low and 32,494 HER2-zero cases. The patient populations were from various regions including the Americas, Asia, and Europe. The majority of studies were published within the last three years. The NOS was leveraged for risk of bias assessment. Among these studies, 32 were classified as high quality and 4 as moderate quality (**Table 1**).

**Table 1:** General characteristics and NOS scores of the included studies.

Author	Publication year	Region	Total sample size (n)	Average age (year)	Follow-up time (month)	Tumor staging (metastatic vs. non-metastatic)	Outcome measures	NOS
Bueno MJ (12)	2024	Spain	459	54.16	67.6	Non-metastatic	RFS	8
Liu X (13)	2024	China	202	51.36	111	Non-metastatic	RFS	9
Liu JJ (14)	2024	China	319	–	–	Non-metastatic	DFS, OS, pCR	8
Atallah NM (15)	2023	UK	630	56.67	60	Non-metastatic	BCSS, DFS	9
Schettini F (16)	2024	Int’l	1983	34.65	87.6	Non-metastatic	DFS, OS	9
Raghavendra AS (17)	2024	US	977	50.5	42	Non-metastatic	RFS, OS, pCR	9
Won HS (18)	2022	Korea	6934	48.52	148	Non-metastatic	BCSS, OS	9
Shao Y (19)	2022	China	87	50.89	–	Non-metastatic	DFS, OS, pCR	8
Schettini F (20)	2021	Spain	706	57.65	90.3	Non-metastatic	OS	9
Shao Y (21)	2024	China	111	50.36	–	Non-metastatic	DFS, OS, pCR	8
Hu S (22)	2024	China	350	–	63.4	Metastatic	PFS, OS	9
De Calbiac O (23)	2022	France	2783	–	49.5	Metastatic	PFS, OS	9
Li Y (24)	2023	China	254	54.6	73	Non-metastatic	BCSS, DFS	8
Yi XL (25)	2024	China	21	52.86	–	Non-metastatic	pCR	4
Shi Z (26)	2024	China	638	–	–	Non-metastatic	DFS, OS, pCR	8
Baez-Navarro X (27)	2024	Neth.	821	–	–	Non-metastatic	pCR	8
Baez-Navarro X (28)	2024	Neth.	2509	–	31.5	Non-metastatic	OS, pCR	8
Baez-Navarro X (30)	2023	Neth.	4987	–	45.6	Non-metastatic	OS	9
de Moura Leite L (29)	2021	Brazil	313	44.95	59	Non-metastatic	RFS, OS, pCR	8
Xu W (31)	2023	China	140	–	24	Non-metastatic	DFS, pCR	7
Ma Y (33)	2024	China	1445	–	55.1	Non-metastatic	BCSS, OS, pCR	9
Schmidt G (32)	2016	GER	1013	–	40.36	Non-metastatic	DFS, OS	9
Schmidt G (34)	2014	GER	121	55.97	–	Non-metastatic	DFS, OS	8
Shi Y (35)	2024	China	191	–	–	Non-metastatic	pCR	8
Domergue C (36)	2022	France	437	51.18	72.9	Non-metastatic	DFS, OS, pCR	9
Li H (37)	2024	China	20029	51.9	59.5	Non-metastatic	OS, pCR	9
Alves FR (38)	2022	PT	32	53.4	35.5	Non-metastatic	pCR	4
Gampenrieder SP (39)	2023	Int’l	691	58.14	–	Metastatic	OS	8
Sanomachi T (40)	2023	Japan	42	60.11	121	Non-metastatic	DFS, OS	6
Tuluhong D (41)	2023	China	58	–	72.7	Non-metastatic	OS	9
Zhao S (42)	2024	China	316	–	57	Non-metastatic	pCR, DFS	8
Tarantino P (43)	2022	US	697	58.99	10	Non-metastatic	pCR	8
Özyurt N (44)	2024	Turkey	620	49.11	31	Non-metastatic	DFS, pCR, OS	9
Park WK (45)	2024	Korea	2542	49.29	62	Non-metastatic	DFS, BCSS, OS, pCR	9
Xu B (46)	2023	China	523	52.53	46.2	Non-metastatic	DFS, OS	8
Jacot W (47)	2021	France	296	58.01	116.4	Non-metastatic	RFS, OS	6

### 3.3 Meta-analysis results

#### 3.3.1 HER2 status and pCR

**Figure 2:**
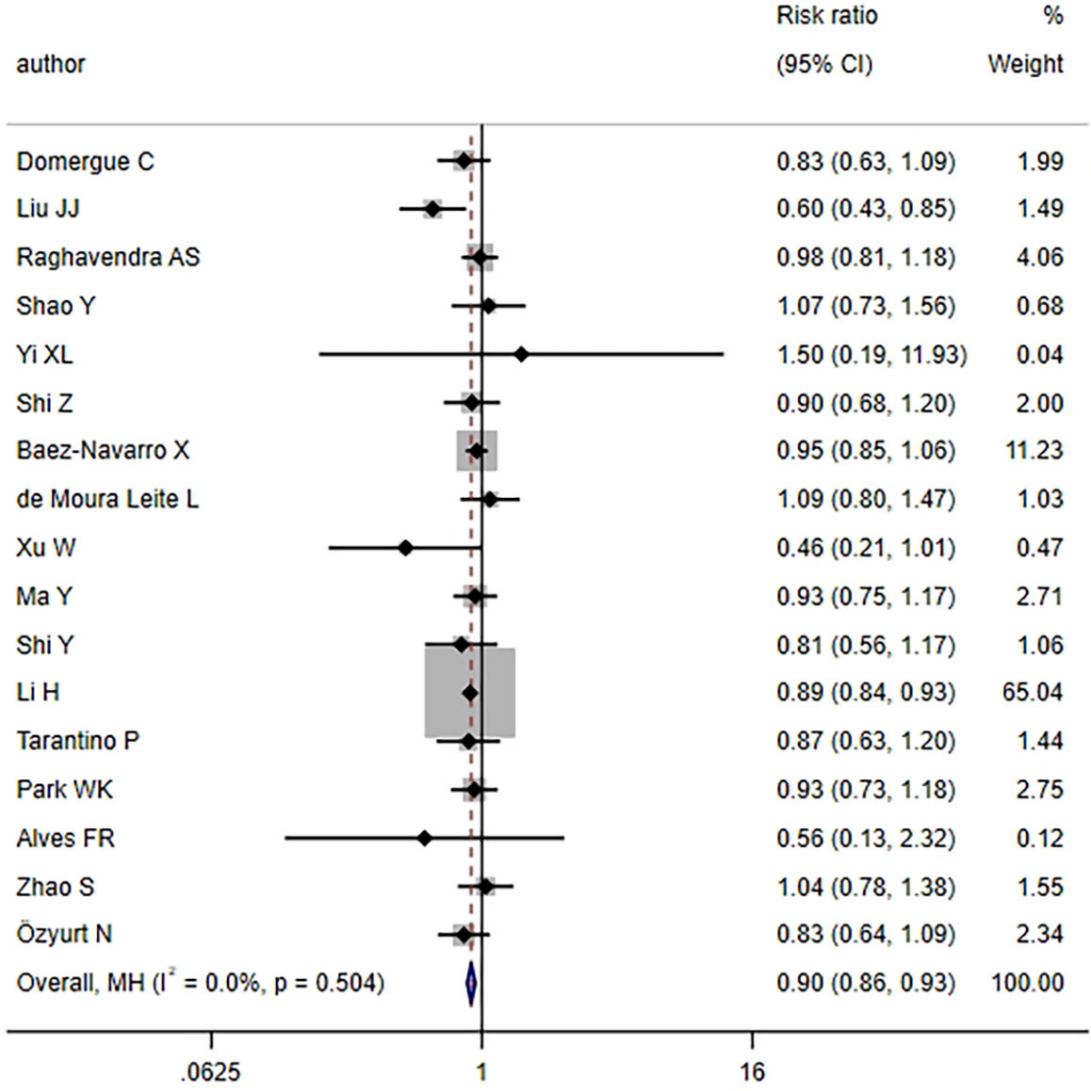
HER2 status and pCR (HER2-low vs. HER2-zero).

17 articles examined the pCR rate in TNBC patients following NAT. The heterogeneity test revealed no notable heterogeneity across studies (*I^2^* = 0.0%, *P* = 0.504) (**Figure 2**), thus the FEM was employed for aggregating effect size. The combined results demonstrated that the HER2-low group exhibited a lower pCR rate relative to the HER2-zero group, with statistical significance (*RR* = 0.90, 95%*CI*: 0.86-0.93, *P <* 0.001). This finding implied that TNBC patients with a HER2-low status might exhibit a slightly inferior response to NAT compared to those with a HER2-zero status.

#### 3.3.2 HER2 status and OS

**Figure 3:**
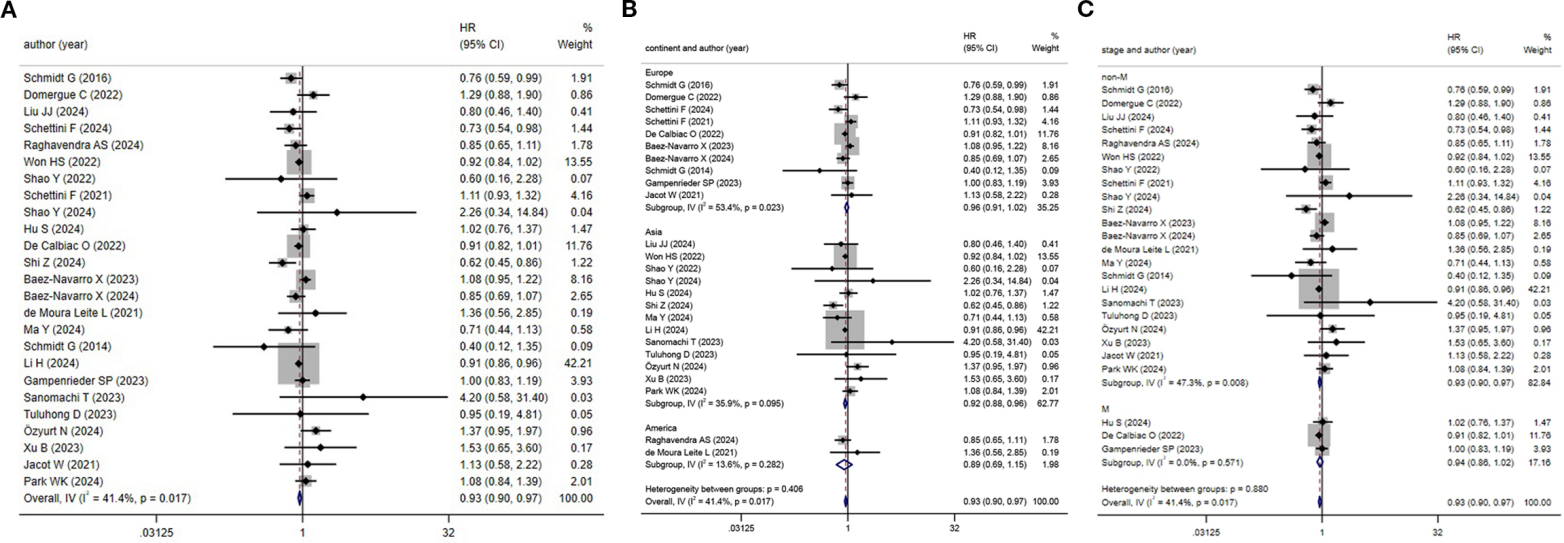
HER2 status and OS (HER2-low vs. HER2-zero) **(A)** HER2 status and OS; **(B)** Subgroup analysis by different continents; **(C)** Subgroup analysis by tumor staging.

25 articles documented the OS rate for TNBC patients. According to the heterogeneity test, a low level of heterogeneity was noted (*I^2^* = 41.4%, *P* = 0.017). Hence, the FEM was leveraged for analysis (**Figure 3A**). Based on the aggregated findings, HER2-low patients experienced a notably enhanced OS relative to those classified as HER2-zero, with statistical significance (*HR* = 0.93, 95%*CI*: 0.90-0.97, *P* < 0.001). Subgroup analysis by different continents demonstrated that in the Asian population, HER2-low patients experienced enhanced OS than HER2-zero patients, with statistical significance (*HR* = 0.92, 95%*CI*: 0.88-0.96, *P* < 0.001). Nonetheless, no statistically significant differences were noted in the European population (*HR* = 0.96, 95%*CI*: 0.91-1.02, *P* = 0.237) and Americas (*HR* = 0.89, 95%*CI*: 0.69-1.15, *P* = 0.368) (**Figure 3B**). The subgroup analysis by tumor staging uncovered that in the population of non-metastatic TNBC, the HER2-low group demonstrated a remarkably enhanced OS relative to the HER2-zero group, and this result was statistically significant (*HR* = 0.93, 95%*CI*: 0.90-0.97, *P* < 0.001). However, no results with statistical significance were noted in the metastatic TNBC population (*HR* = 0.94, 95%*CI*: 0.86-1.02, *P* = 0.153) (**Figure 3C**).

#### 3.3.3 HER2 status and DFS

**Figure 4:**
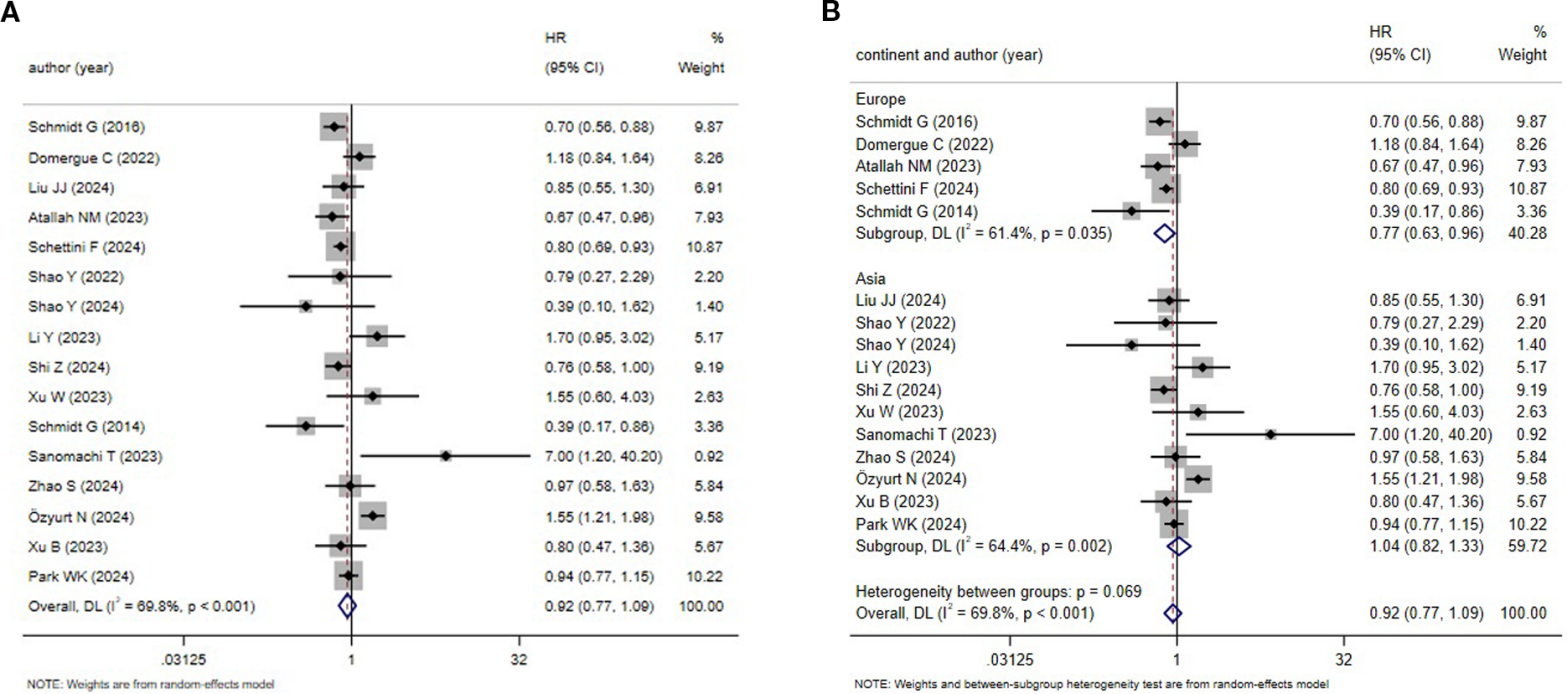
HER2 status and DFS (HER2-low vs. HER2-zero) **(A)** HER2 status and DFS;**(B)** Subgroup analysis by different continents.

16 studies explored the DFS rate for TNBC patients. A relatively high level of heterogeneity was noted (*I^2^* = 69.8%, *P* < 0.001), leading to the use of the REM (**Figure 4A**). According to the meta-analysis, HER2-low patients might experience an improved prognosis. Nonetheless, no statistical significance was noted (*HR* = 0.92, 95%*CI*: 0.77-1.09, *P* = 0.338). The subgroup analysis uncovered that different continents were not a source of research heterogeneity (**Figure 4B**).

#### 3.3.4 HER2 status with BCSS and RFS

**Figure 5:**
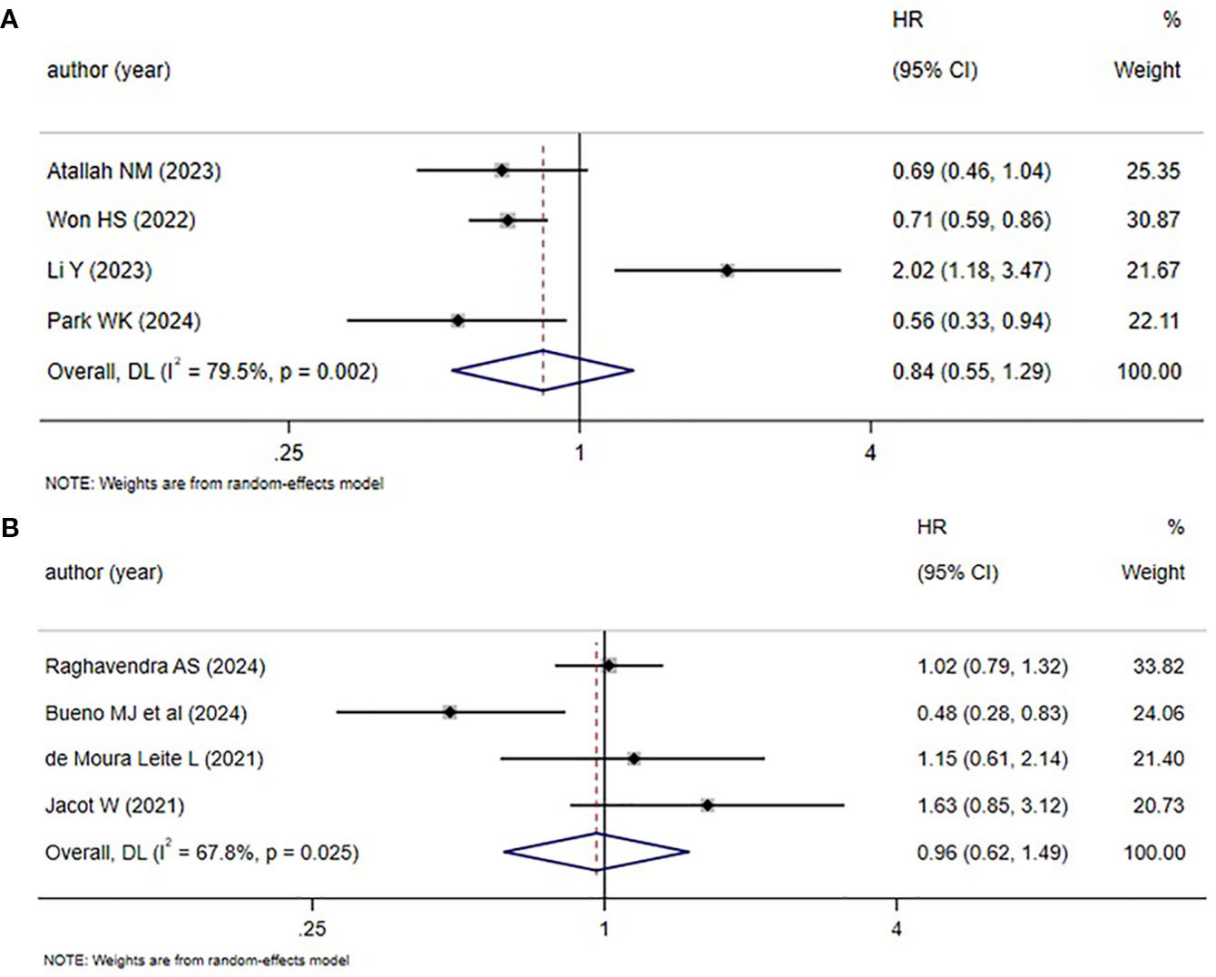
HER2 status and BCSS as well as RFS (HER2-low vs. HER2-zero) **(A)** HER2 status and BCSS; **(B)** HER2 status and RFS.

Four studies each reported the BCSS and RFS rates in TNBC patients. Heterogeneity analyses both revealed notable heterogeneity in studies (BCSS: *I^2^* = 79.5%, *P* = 0.002; RFS: *I^2^* = 67.8%, *P* = 0.025). Thus, all effect sizes were combined using the REM (**Figures 5A, B**). The results uncovered that the HER2-zero status was linked to worse BCSS and RFS. However, neither result showed statistical significance (BCSS: *HR* = 0.84, 95%*CI*: 0.55-1.29, *P* = 0.424; RFS: *HR* = 0.96, 95%*CI*: 0.62-1.49, *P* = 0.862). Therefore, the link between HER2 status and BCSS and RFS needed to be further studied.

### 3.4 Sensitivity analysis and publication bias

**Figure 6:**
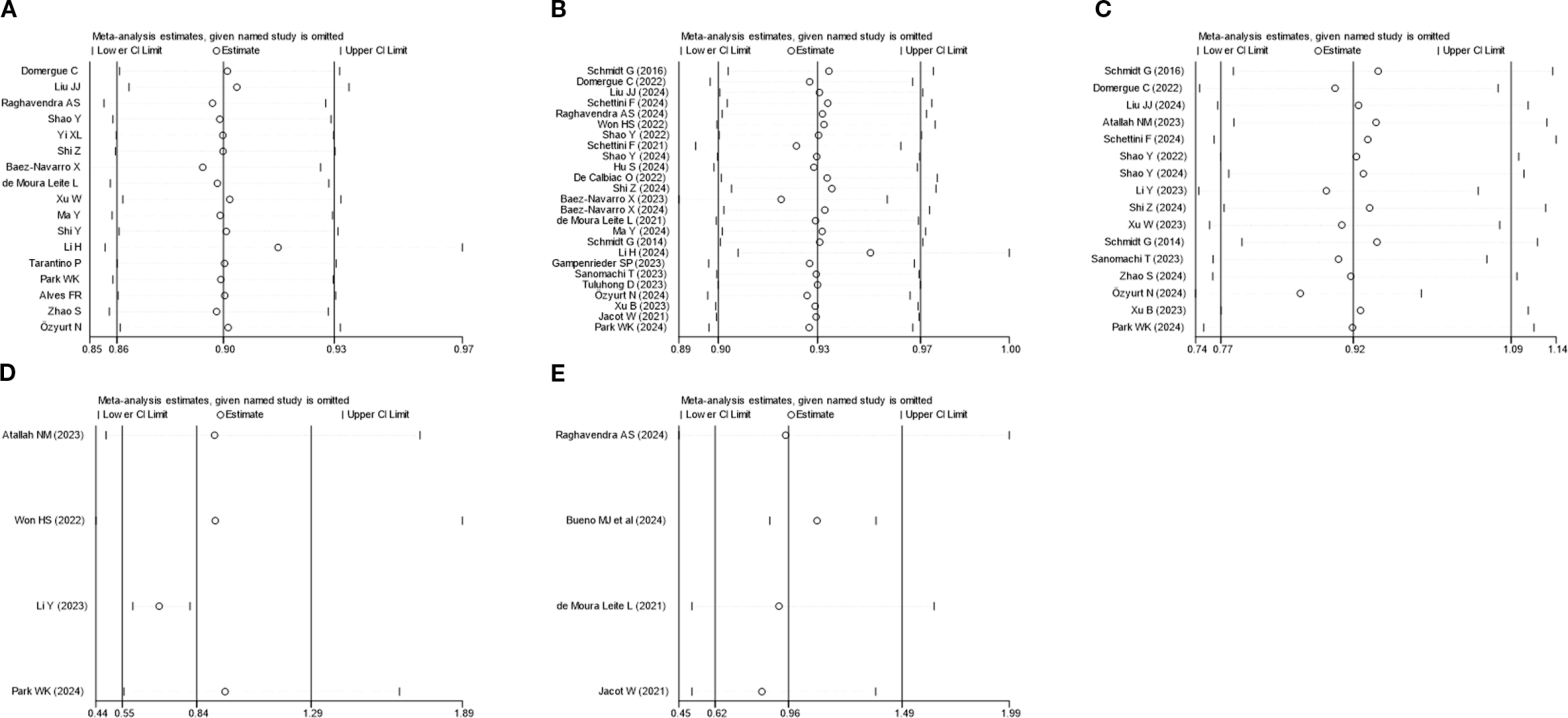
Sensitivity analysis **(A)** pCR; **(B)** OS; **(C)** DFS; **(D)** BCSS; **(E)** RFS.

**Figure 7:**
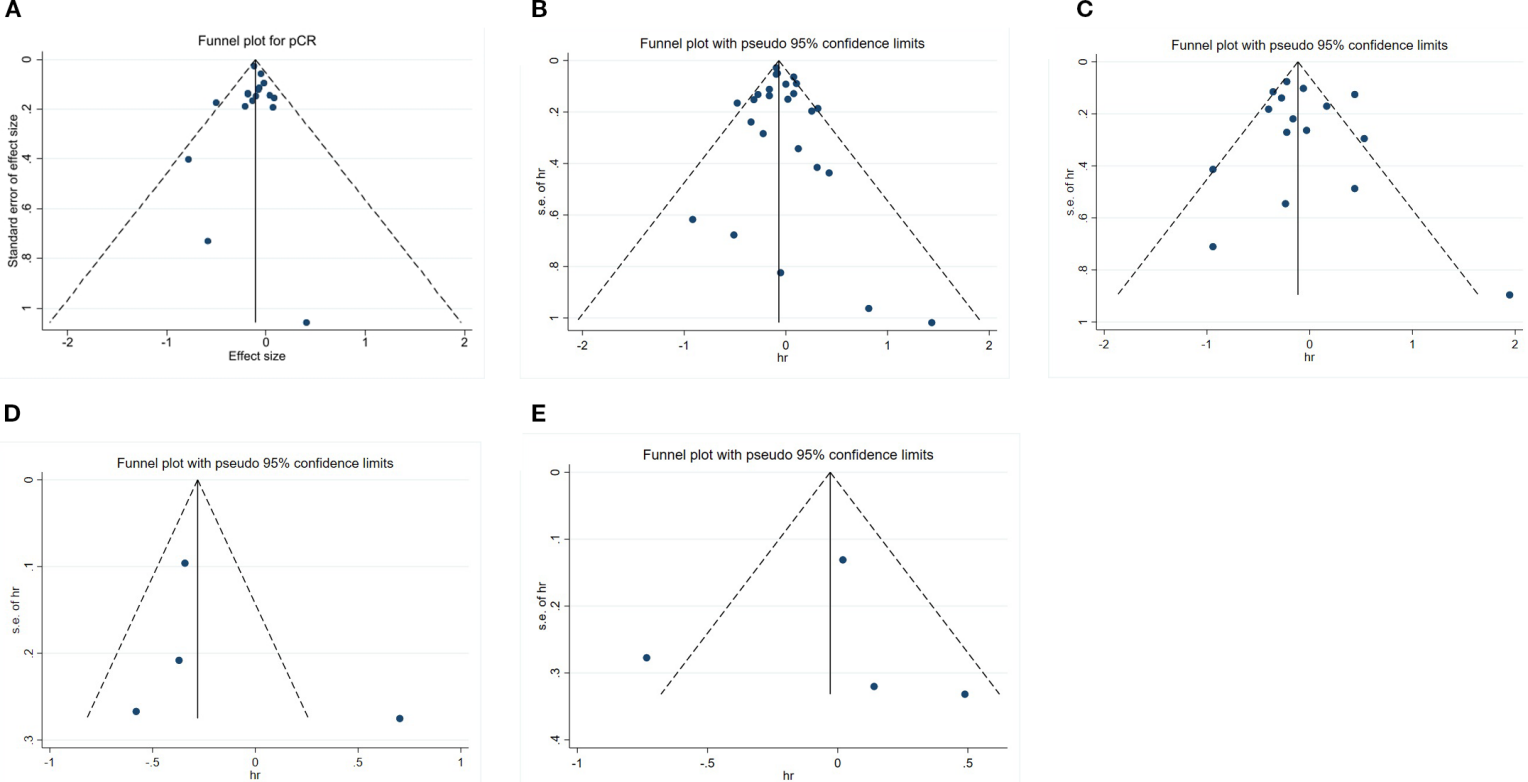
Funnel plots for publication bias. **(A)** pCR; **(B)** OS; **(C)** DFS; **(D)** BCSS; **(E)** RFS.

Sensitivity analysis was executed utilizing the leave-one-out method for PCR, OS, DFS, BCSS, and RFS. The results uncovered that none of the studies markedly impacted the combined effect size, indicating a high level of stability (**Figure 6**). Publication bias was analyzed for studies on each outcome indicator. Egger’s test revealed that all *P-*values were above 0.05, indicating no publication bias (**Figure 7**). The results of the meta-analysis were reliable.

## 4 Discussion

BC is one of the most widespread malignant tumors in women across the globe. TNBC, as a subtype with strong invasiveness and poor prognosis, has remained a challenge in clinical treatment. In recent years, with the in-depth study of the biological characteristics of TNBC, the role of HER-2 status in TNBC has attracted much attention. This study conducts a systematic review and meta-analysis of 36 cohort studies to examine the influence of HER2-low and HER2-zero status on pCR and survival rates in TNBC patients. Our findings reveal that the HER2 status noticeably impacts the prognosis of TNBC patients, particularly concerning pCR and OS. Subgroup analysis further elucidates the variability of this effect across different regional populations.

This study finds that among TNBC patients receiving NAT, the HER2-zero group exhibits a greater pCR rate. This finding indicates that TNBC patients with HER2-zero status may exhibit a more favorable response to NAT. This aligns with the findings from multiple studies. For instance, A S Raghavendra et al. (17) also indicate that TNBC patients with HER2-zero status have a greater likelihood of achieving a pCR following NAT. This might be tied to the biological characteristics of TNBC, as tumor cells that do not express HER2 may exhibit greater sensitivity to chemotherapeutic agents. The mechanism may include the following aspects. First, tumor proliferation and grade may be a pivotal factor. The study by Yue Shi et al. (35) has shown that in HER2-zero TNBC, patients with positive androgen receptor and high Ki-67 expression have a significantly higher pCR rate, suggesting that hormone receptor signaling pathways and cell proliferation activity may jointly regulate chemotherapy sensitivity. Another study (13) has further pointed out that compared with the HER2-low group, the Ki-67 expression level and histological grade in HER2-zero TNBC are higher and are associated with a higher pCR rate. Second, molecular mutation profile may be another mechanism. A multicenter retrospective cohort study (48) has found that PI3K/AKT pathway activation and BRCA-like phenotypes are significantly enriched in the HER2-zero subtype, suggesting that this subtype may have higher genomic instability or more DNA repair defects, thereby enhancing sensitivity to platinum-based chemotherapy drugs. Third, immune microenvironment may also play a crucial role. Studies (49, 50) have shown that HER2-low TNBC may inhibit antigen presentation through HLA gene hypermethylation, reducing immune cell infiltration within the tumor and forming an immune escape microenvironment. This may be a key mechanism for the poor efficacy of neoadjuvant chemotherapy.

However, Helal et al. have pointed out that TNBC patients with a HER2-low status can achieve a relatively high pCR under certain specific treatment regimens. This implies that the influence of HER2 status on treatment response in TNBC patients might vary depending on the regimen employed (51). This difference might be linked to the geographical distribution of study populations and variations in treatment protocol selection. In addition, the detection methods and standards for HER2 status vary across studies. This may also contribute to the inconsistent findings observed across the studies. Currently, the detection of HER2 status relies on IHC and ISH techniques. Nevertheless, these methods might have limitations in detecting low HER2 expression (52). Further research should optimize HER2 detection methods and establish unified testing standards to enhance the comparability and reliability of research results.

In terms of OS, this study points out that TNBC patients with HER2-low status experience an improved prognosis relative to those with HER2-zero, indicating that the HER2-low status might be tied to a longer survival time. Another study has indicated that TNBC patients with HER2-low status exhibit higher survival rates during long-term follow-up (53). Given the vast differences in pCR and OS outcomes between HER2-low and HER2-zero subtypes, most current studies focus on exploring the mechanisms from the following perspectives. First, from the perspective of tumor proliferation activity and histological grade, HER2-low TNBC typically exhibits a lower Ki-67 index and lower histological grade. Although its pCR rate after neoadjuvant chemotherapy is lower than that of HER2-zero, HER2-low has a lower risk of long-term recurrence due to its weaker tumor invasiveness and metastasis ability. This survival advantage is even more significant in chemotherapy-resistant patients (13, 54). Second, Their difference in survival outcomes may also be explained by the potential efficacy of targeted therapy. ERBB2 gene expression is upregulated in HER2-low TNBC, activating the downstream HER2 signaling pathway, making it a potential target for antibody-drug conjugates (ADCs) such as T-DXd. Traditional anti-HER2 monoclonal antibodies are ineffective against HER2-low tumors, while ADCs can effectively kill tumors through a bystander effect. This mechanism is consistent with the results of the DESTINY-Breast04 trial in HER2-low metastatic breast cancer (26, 50, 53, 55).

Certainly, some studies suggest that the influence of HER2 status on OS might not be noticeable (26). This discrepancy could be related to variations in sample size, population characteristics, and treatment protocols among the included studies. Insufficient sample size might lead to inadequate statistical power, thereby obscuring the true impact of HER2 status on OS. The subgroup analyses across different continents indicate various results, suggesting that regional differences might be a potential factor influencing the link between HER2 status and OS. The subgroup analysis by tumor staging demonstrates that the results for the non-metastatic TNBC population are consistent with those of the overall population. In contrast, this phenomenon is not observed in the metastatic TNBC group. This implies that variations in research design, treatment protocols, and baseline characteristics of patients across different regions might notably influence the results. Therefore, further research should consider conducting multicenter studies in various regions to expand the sample size. Additionally, subgroup analyses should be performed on different populations and treatment regimens to provide higher quality evidence.

This study implies that HER2-low TNBC patients might have better DFS, BCSS, and RFS outcomes, although the results are not statistically significant. This aligns with some previous studies (56, 57). Some studies reveal no notable connection of HER2 status with DFS, BCSS, and RFS (43, 58). This difference may be linked to the biological characteristics of HER2-zero tumor cells, which may exhibit enhanced tumor stem cell properties or a greater capacity for immune evasion (57). Furthermore, the evaluation of DFS, BCSS, and RFS is affected by various factors, including the selection of treatment protocols, individual patient differences, and the duration of follow-up. Some studies may have employed more effective chemotherapy regimens or combination treatment strategies, thereby obscuring the true impact of HER2 status. Moreover, the insufficient sample size and relatively short follow-up duration may result in inadequate statistical power, preventing the detection of notable differences between HER2 status and the three outcome measures. Therefore, subsequent research should expand the sample size, standardize treatment protocols, and extend follow-up duration. This would further clarify the role of HER2 status in TNBC prognosis and provide more precise guidance for the clinical treatment of TNBC patients.

This study, through a systematic search and rigorous selection across multiple databases, has incorporated a substantial number of high-quality cohort studies with considerable sample sizes. Employing diverse statistical analysis methods—including heterogeneity testing, subgroup analyses, and sensitivity analyses—this research ensures the reliability and stability of its findings. These findings provide robust references for the prognosis of TNBC patients.

However, certain limitations still exist. (i) Despite a rigorous quality assessment of the included studies, some may still be subject to bias, potentially affecting the results of this research. (ii) The number of studies on certain outcome indicators (like BCSS and RFS) is limited, potentially resulting in insufficient statistical power for the findings. (iii) Due to the high heterogeneity of TNBC, the prognostic influence of HER2 status might be affected by individual patient differences, treatment regimens, and tumor biological characteristics. However, the data from the included studies are inadequate to support further analysis of these influencing factors. (iv) This study only includes English studies, which may result in omitting important non-English research.

In conclusion, this study reveals that the HER2 status evidently influences the prognosis of TNBC patients, particularly in terms of pCR and OS. Patients with HER2-zero status have a greater likelihood of achieving a pCR following NAT, whereas those with HER2-low status may exhibit better long-term survival outcomes. Based on this conclusion, HER2-low-expressing breast cancer may be considered as an independent subtype of TNBC in future studies. However, this impact exhibits certain discrepancies across different studies. This might be tied to factors like treatment protocols, sample size, and follow-up duration. Future research should integrate a broader range of clinical factors and biological markers, optimize the detection methods for HER2 status, employ multi-omics technologies, and conduct multicenter studies. This comprehensive approach aims to further explore the link of HER2 status with prognosis in TNBC patients, ultimately guiding personalized precision treatment strategies for this patient population. Moreover, for TNBC patients with HER2-low status, future research may delve into their responses to various therapeutic regimens. For example, TNBC patients with low HER2 expression may be included as an independent group in clinical trials of HER2-targeted therapy, with the aim of devising more efficacious treatment strategies.

## Data Availability

The original contributions presented in the study are included in the article/supplementary material. Further inquiries can be directed to the corresponding author.
